# Absence of Mycoplasma Contamination in the Anthrax Vaccine

**DOI:** 10.3201/eid0801.010091

**Published:** 2002-01

**Authors:** Mary Kate Hart, Richard A. Del Giudice, George W. Korch

**Affiliations:** * United States Army Medical Research Institute of Infectious Diseases, Fort Detrick, Frederick, Maryland, USA; † National Cancer Institute-Frederick Cancer Research and Development Center, Frederick, Maryland, USA

**Keywords:** anthrax, vaccine, mycoplasma

## Abstract

Mycoplasma contamination of the licensed anthrax vaccine administered to military personnel has been suggested as a possible cause of Persian Gulf illness. Vaccine samples tested by nonmilitary laboratories were negative for viable mycoplasma and mycoplasma DNA and did not support its survival. Mycoplasma contamination of anthrax vaccine should not be considered a possible cause of illness.

Anthrax Vaccine Adsorbed (AVA, BioPort Corporation, Lansing, MI) is a licensed vaccine for anthrax that was administered to approximately 150,000 U.S. military personnel during the Persian Gulf War. It was used more recently as part of a comprehensive vaccination policy for Department of Defense (DOD) service members. The vaccine, which is administered subcutaneously over an 18-month schedule, is derived from sterile, acellular filtrates of microaerophilic cultures of the avirulent, nonencapsulated V770-NP1-R strain of *Bacillus anthracis*. The cultures are grown in a sterile synthetic liquid medium devoid of enriched supplements such as serum. The filtrates predominantly contain the protective antigen of anthrax. The final vaccine formulation contains protective antigen adsorbed to aluminum hydroxide (<2.4 mg/0.5 mL) as an adjuvant. Formaldehyde (<0.02%) is added as a stabilizer and benzethonium chloride (0.0025%) as a preservative. The final product is checked for potency and safety according to U.S. Food and Drug Administration (FDA) regulations.

Approximately 1.9 million doses of AVA were administered in the United States from January 1990 through August 2000 (*1*), with 1,544 adverse reactions (0.08% of all injections) reported through the Vaccine Adverse Event Reporting System (*2*). Most of these adverse reactions were limited to the injection site; local hypersensitivity, edema, and pain were the most commonly reported, although 76 (4.9%) of the adverse reactions were classified as serious.

## The Study

Recently, Nicolson et al. (*3*) suggested that contamination of this vaccine with *Mycoplasma fermentans* could have been responsible for human illness specifically associated with Persian Gulf syndrome. Mycoplasma contamination is considered to be a potential problem for vaccines that are produced in cell cultures. Such vaccines have to meet specific FDA testing requirements to demonstrate the absence of mycoplasma contamination, which is considered unlikely in vaccines that are not cell culture-derived, such as AVA,. However, in response to the continuing controversy surrounding this vaccine, the possibility of its being contaminated with mycoplasma was examined by two different techniques.

AVA was administered to U.S. personnel during Operation Desert Storm/Desert Shield (ODS/DS), including lots FAV 001 through FAV 007. Although the lots tested in this study were not administered to U.S. personnel deployed during ODS/DS, the filling and potency testing of lot FAV 008 in March 1991 were contemporaneous with that of FAV 007 and were subject to the same regulatory criteria for lot release and use. Its use in the current vaccination program was permitted through shelf life extension based on satisfactory potency testing.

Twenty vials of AVA, including samples from four lots, were obtained from eight DOD vaccination clinics across the United States (Table). The tested lots represented all unexpired vaccine available to the DOD at the time of testing. Testing of unexpired material was deemed necessary to avoid potential uncertainty regarding degradation of vaccine components or analytes.

**Table:** Anthrax Vaccine Adsorbed samples evaluated for mycoplasma contamination

Vaccine lot Expiration date	Sites providing vaccine^a^
FAV048B 13 Apr 2002	355 Medical Squadron, Davis Monthan Air Force Base, AZ N.R.C., Kansas City, MO Camp Pendleton USMC
FAV047 8 Sep 2001	USAMRIID, Ft Detrick , MD (four vials) Pearl Harbor NMC
FAV031 6 Oct 2000	Ft. Worth Base Naval Clinic Pentagon Clinic
FAV008 5 Aug 2000	Davis Monthan Air Force Base, AZ 169th FW/Base Supply, McEntire ANG Station, Eastover, SC

^a^Vaccine vials were shipped to Fort Detrick on cold packs. Two vials from each lot were sent unless noted. Two vials of lot FAV047 from USAMRIID were sent to each testing laboratory as a control.

These vials were divided into two matched sets and were sent to two nonmilitary laboratories for testing for mycoplasma contamination. One set was tested by culture techniques for the presence of live organisms at the Mycoplasma Laboratory, Science Applications International Corporation, National Cancer Institute, Frederick Research and Development Center, Frederick, MD. A commercial nongovernment facility, Charles River Tektagen, tested the second set for mycoplasma DNA by polymerase chain reaction (PCR) assay.

Ten anthrax vaccine vials were tested for the presence of mycoplasma by standardized cultivation methods (*4*–*6*) with three different media. The media used for isolation were SP-4 (*4*), DM-1 (*5*), and M-CMRL (*6*); all three supported the growth of *Mycoplasma* organisms. Vaccine samples were removed under Biosafety Level-2 conditions and tested both undiluted and at a 10-fold dilution to reduce the concentrations of stabilizer and preservative. Each culture plate was inoculated with 0.1 mL of vaccine. M-CMRL plates were incubated aerobically at 36°C, and SP-4 and DM-1 plates were incubated anaerobically at 36°C. After 10 days, all plates were examined microscopically. No evidence of *Mycoplasma* colonies was found in any of the culture plates inoculated with the AVA samples.

To determine if *Mycoplasma* organisms could survive in the vaccine, which contains formaldehyde and benzethonium chloride, a fifth lot of vaccine (FAV038) was obtained and evaluated. This vaccine lot also tested negative. To test the ability of *Mycoplasma* to survive in the vaccine, *M. fermentans* strain incognitus (*7*) was inoculated at a concentration of 1.54 x 10^8^ CFU per 0.1 mL into 5 mL of vaccine and mixed. Eight serial 10-fold dilutions of this “time 0" sample were immediately plated on SP-4 agar plates. The remaining vaccine-*Mycoplasma* mixture was held at 4°C and sampled at 24, 48, and 72 hours. Tenfold serial dilutions of these sequential samples were immediately plated as described for the time 0 sample.

All SP-4 plates were incubated for a minimum of 10 days and examined for viable colonies. The colony growth on the time 0 plates was atypical, with much debris on the low dilution plates. The titer of mycoplasma from the time 0 sample was 4.2 x 10^5^ CFU per 0.1 mL. Inactivation of the organisms by the preservatives in the vaccine was rapid, as no growth was detected on plates inoculated with samples taken from the mycoplasma-vaccine mixtures held at 4°C for 24, 48, or 72 hours.

Testing for the presence of mycoplasma DNA in the second set of AVA samples, representing four lots, was performed by a commercial testing facility (Charles River Tektagen, Malvern, PA) that used the Detection of Mycoplasma by PCR kit (#90-100K, American Type Culture Collection, Rockville, MD). Samples were tested first for the presence of any mycoplasma DNA; the species was then determined for samples that tested positive. The vaccine samples were centrifuged (12,000 x *g* for 10 minutes at 4°C) and processed for DNA extraction according to the kit’s instructions. Samples were amplified in a nested PCR reaction and examined by gel electrophoresis. The positive control samples were *M. pirum* and *Acholeplasma laidlawii*, and the negative control samples were mycoplasma broth, Hut78 cell extract, and sterile RNase-free water. All 10 of the tested AVA samples were negative for the presence of mycoplasma DNA (Figure).

**Figure:**
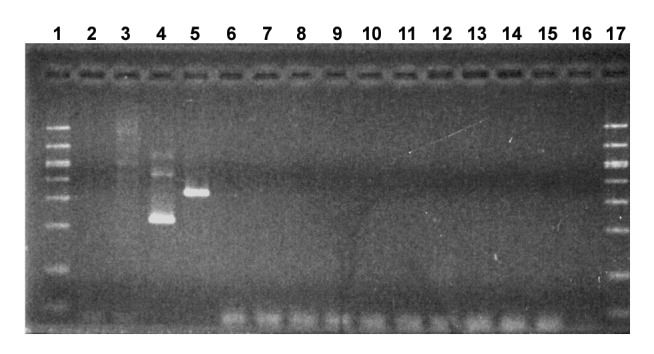
Evaluation of Anthrax Vaccine Adsorbed (AVA) for amplified mycoplasma DNA by gel electrophoresis. Molecular weight markers were run in lanes 1 and 17. Control samples in lanes 2-5 were mycoplasma broth, Hut 78 cell extract, *Acholeplasma laidlawii*, and *Mycoplasma pirum*, respectively. The AVA samples were in lanes 6 to 15: Lot FAV048B from Davis Monthan AFB (lane 6), Kansas City MO NRC (lane 7), and Camp Pendleton (lane 8); Lot FAV047 from Fort Detrick (lanes 9 and 11) and Pearl Harbor NMC (lane 10); Lot FAV031 from Fort Worth Base Naval Clinic (lane 12) and the Pentagon Clinic (lane 13); and Lot FAV008 from Davis Monthan AFB (lane 14) and McEntire ANG Station (lane 15). Lane 16 contained water. Bands seen below 100 base pairs are primer multimers.

## Conclusions

No evidence was found either from culture or mycoplasma-specific nucleic acid amplification methods to suggest that a mycoplasma contaminant was present in the vaccine lots tested. These results are consistent with those of a previous report that found no evidence of such contamination in another anthrax vaccine (*8*). Additionally, *Mycoplasma* organisms deliberately added to AVA did not survive for even 1 day, presumably because of the preservatives added to the vaccine formulation to retard adventitious agent growth.

These results argue against assertions that this vaccine was contaminated with *Mycoplasma* organisms and that such putative contamination contributed to human illness. This finding is consistent with a serologic study of pre- and post-Gulf War serum samples from symptomatic and asymptomatic military personnel, which found no evidence of an association between Gulf War illness and infection with *M. fermentans* (*9*).
